# Temporal Patterns of Abundance of *Aedes aegypti* and *Aedes albopictus* (Diptera: Culicidae) and Mitochondrial DNA Analysis of *Ae. albopictus* in the Central African Republic

**DOI:** 10.1371/journal.pntd.0002590

**Published:** 2013-12-12

**Authors:** Basile Kamgang, Carine Ngoagouni, Alexandre Manirakiza, Emmanuel Nakouné, Christophe Paupy, Mirdad Kazanji

**Affiliations:** 1 Institut Pasteur de Bangui, Bangui, Central African Republic; 2 Centre International de Recherches Médicales de Franceville, Franceville, Gabon; 3 Laboratoire des Maladies Infectieuses et Vecteurs: Écologie, Génétique, Évolution et Contrôle, UMR 224-5290, CNRS-IRD-UM1-UM2, IRD Montpellier, France; Johns Hopkins Bloomberg School of Public Health, United States of America

## Abstract

The invasive Asian tiger mosquito *Aedes albopictus* (Diptera: Culicidae) was first reported in central Africa in 2000, in Cameroon, with the indigenous mosquito species *Ae. aegypti* (Diptera: Culicidae). Today, this invasive species is present in almost all countries of the region, including the Central African Republic (CAR), where it was first recorded in 2009. As invasive species of mosquitoes can affect the distribution of native species, resulting in new patterns of vectors and concomitant risk for disease, we undertook a comparative study early and late in the wet season in the capital and the main cities of CAR to document infestation and the ecological preferences of the two species. In addition, we determined the probable geographical origin of invasive populations of *Ae. albopictus* with two mitochondrial DNA genes, *COI* and *ND5*. Analysis revealed that *Ae. aegypti* was more abundant earlier in the wet season and *Ae. albopictus* in the late wet season. Used tyres were the most heavily colonized productive larval habitats for both species in both seasons. The invasive species *Ae. albopictus* predominated over the resident species at all sites in which the two species were sympatric. Mitochondrial DNA analysis revealed broad low genetic diversity, confirming recent introduction of *Ae. albopictus* in CAR. Phylogeographical analysis based on COI polymorphism indicated that the *Ae. albopictus* haplotype in the CAR population segregated into two lineages, suggesting multiple sources of *Ae. albopictus*. These data may have important implications for vector control strategies in central Africa.

## Author Summary

*Aedes aegypti* and *Ae. albopictus* are the main vectors of human arboviral diseases such as dengue and chikungunya. *Ae. aegypti* is indigenous in the Central African Republic (CAR), whereas *Ae. Albopictus*, originating from Asian forests, was first reported in 2009. To determine the consequences of this invasion of *Ae. albopictus* for epidemiological transmission of arboviruses, we conducted a comparative study in the early and late wet season in the capital, Bangui, and in the other main cities of the country to document infestation by the two species and their ecological preferences. In addition, we explored the geographical origin of populations of *Ae. albopictus* with two mitochondrial DNA genes (*COI* and *ND5*). We demonstrate that *Ae. aegypti* predominates early and *Ae. albopictus* late in the wet season. *Ae. albopictus* was the most prevalent species in almost all the sites investigated, except Bouar, where only *Ae. aegypti* was found, suggesting that *Ae. albopictus* tends to supplant *Ae. aegypti* in sympatric areas. Mitochondrial DNA analysis revealed broad low genetic diversity, confirming recent introduction of *Ae. albopictus*. Phylogeographical analysis with MtDNA *COI* gene suggested that *Ae. albopictus* in CAR came from multiple invasions and from multiple population sources.

## Introduction

*Aedes aegypti* Linneaus 1762 and *Ae. albopictus* Skuse 1894, two mosquitoes belonging to the *Stegomyia* subgenus, are the main epidemic vectors of dengue and chikungunya viruses worldwide [1], [2], [3], [4]. Both species are established in sub-Saharan Africa, where *Ae. aegypti* is native [5]. *Ae. albopictus* originated in Asia [6] and has invaded Europe, the Americas and Africa during the past three decades. This rapid global spread was favoured by international trade, especially of used tyres [7], and by the differing physiology and ecology of many populations, which allows the species to thrive in a wide range of climates and habitats [8]. Since 2000, *Ae. albopictus* has invaded several central African countries, including Cameroon [9], Gabon [10], Equatorial Guinea [11] and the Central African Republic (CAR) [12], where it occurs in human-dominated environments previously colonized by *Ae. aegypti*. Recently, the density of *Ae. albopictus* has reached levels compatible with arbovirus transmission. *Ae. albopictus* is suspected to have played a major role in the transmission of chikungunya virus in Cameroon in 2006 [13] and was shown to be the main vector of both chikungunya and dengue virus in Gabon in 2007 and 2010 [1], [14], [15]. In addition, *Ae. albopictus* populations in Cameroon were shown to be orally susceptible to dengue-2 virus and highly competent for chikungunya virus [1]. It is therefore likely that this invasive mosquito also played a significant role in the chikungunya outbreak in the Republic of Congo in 2011 [16].

Coexistence of *Ae. aegypti* and *Ae. albopictus* has been documented in several regions in the world, where the larvae sometimes share common developmental sites [17], [18], [19], [20]. In areas of South America and South-East Asia where the two species are sympatric, they segregate into different habitats on the basis of environmental factors [17], [21], [22]. *Ae. aegypti* usually dominates in densely crowded urban areas, whereas *Ae. albopictus* dominates in suburban or rural areas. Nevertheless, *Ae. albopictus* can also colonize urban habitats, especially when *Ae. aegypti* is absent [23]. Overlap in the spatial distribution of the two species is thought to result in competitive interaction. Displacement of *Ae. aegypti* after invasion by *Ae. albopictus* was documented in south-eastern USA and Brazil [24], [25], [26] and was suspected in Réunion and Mayotte [27], [28], [29]. Conversely, in Asia, *Ae. aegypti* has an overall competitive advantage over *Ae. albopictus*, especially in urban areas [16], [30], [31]. Although the outcome of competitive interactions between these two species has not yet been studied in Africa, studies in Cameroon showed that invasion by *Ae. albopictus* led to replacement of the native species *Ae. aegypti* in cities in which both species are present [18], [20].

Several phylogeographical studies have been undertaken to determine the origin of invasive populations of *Ae. albopictus* with isoenzymatic and mitochondrial markers. Recent studies with mitochondrial markers challenged the hypothesis of a common origin of North and South American populations [32], and it was suggested that the Brazilian populations were related to South-East Asian rather than temperate Asian populations [33]. Other analyses based on *COI* polymorphism indicate that *Ae. albopictus* populations in Cameroon are related to tropical rather than temperate or subtropical out-groups [34].

Invasion of central Africa by *Ae. albopictus* genetically competent for dengue or chikungunya virus [1] and subsequent modification of *Aedes* populations might affect the epidemiology of these two viruses and lead to major outbreaks. The control of such diseases is based on entomological surveillance and vector control and requires extensive background information on the biology of the mosquito vectors involved. In addition, as the biological traits of mosquitoes are genetically determined [35] and as these traits may influence virus transmission in newly colonized areas, it is important to determine the geographical origin of invading populations. We undertook a study to assess the extent of infestation by *Ae. aegypti* and *Ae. albopictus* in Bangui, the main urban area of CAR, and nearby localities, focusing on larval habitats and spatial distribution. We also explored the phylogenetic relations between the *Ae. albopictus* populations colonizing CAR and out-group populations sampled worldwide.

## Materials and Methods

### Ethics statement

Institutional clearance for this study, including the sampling of mosquitoes, was approved by the national ethical and scientific committees in charge of validating study designs in CAR. For entomological investigation performed on private land or in private residences, all owners or residents gave permission for the study to be conducted.

### Study sites

**Figure 1:**
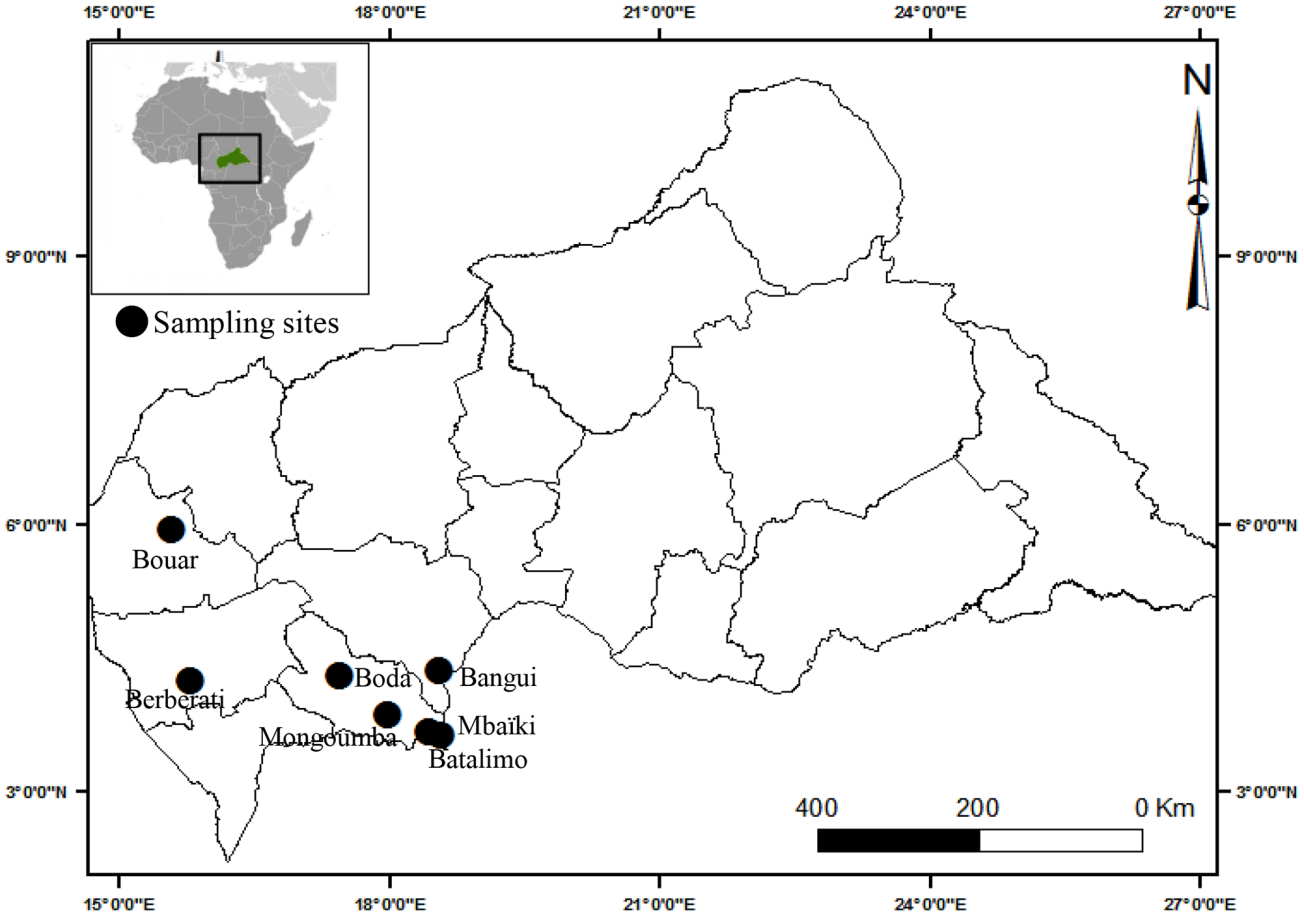
Location of mosquito sampling sites in the Central African Republic.

Mosquitoes were collected between April and November 2012 at seven localities in southern CAR: Mbaïki (3°52N,17°59E), Batalimo (3°40N, 18°27E), Mongoumba (3°38N, 18°35E), Boda (4°18N, 17°27E), Berberati (4°15N, 15°47E), Bouar (5°56N, 15°35E) and Bangui (04°21N, 18°33E) (Figure 1). The surveys were limited to this part of the country as it was the only part that was safe and accessible. The larval ecology of *Ae. aegypti* and *Ae. albopictus* was characterized in Bangui, the capital, with a population of about 900 000. The city is located on the right bank of the Ubangi River, which forms the border between CAR and the Democratic Republic of the Congo. Bangui comprises two blocks: the centre is modern, with urban buildings from the pre-independence period, while the suburbs are unplanned and sparsely populated. The climate is of the Guinean forest type, with alternation of two seasons: a rainy season from March to mid-December and a dry season from mid-December to February. The average annual rainfall is 1543 mm, and the minimum and maximum temperatures are around 15°C and 38°C, respectively.

### Sampling and entomological surveys of immature stages

We undertook ecological characterization of *Ae. aegypti* and *Ae. albopictus* in Bangui and assessed the current spatial distributions of the two species in the southern part of the country. In Bangui, entomological surveys were carried out twice, in April and October 2012, corresponding to early and later in the wet season, respectively. Surveys were undertaken in clusters of houses sampled randomly, each cluster consisting of 10 houses per quarter in each of eight boroughs. In the field surveys, each selected house was geo-referenced with a GPS and visited to record all natural and artificial containers of water (potential containers) and those containing immature stages (larvae and pupae) of *Ae. aegypti* and *Ae. albopictus* (positive containers). Whenever they were present, immature stages were collected for further counting and identification in the insectarium at the Institut Pasteur of Bangui. Positive larval development sites were also geo-referenced, and the type of container, the container volume, the volume, source, use and quality (clear, tinted, organic matter) of water, the presence of plant debris inside the container, the presence of vegetation around the container and sun exposure were noted, with the number of inhabitants per house. On the basis of the nature, the source and the use of the water, potential containers were classified into domestic, peri-domestic and natural. Domestic containers were defined as human-filled receptacles, whereas peri-domestic (e.g. discarded containers) and natural receptacles (e.g. rock and tree holes, leaf axils, empty shells and nuts) were those filled by rain. Larvae and pupae were returned to the insectaries and isolated from predators such as *Culex (Lutzia) tigripes* larvae, counted (larvae L3-4 and pupae), reared to adults and then identified from morphological identification keys [36], [37]. The number of immature stages of each species was estimated from the proportion of emerging adults of each species.

At sites other than Bangui, the surveys were undertaken only in the late wet season. Entomological investigation consisted of a complete inventory of potential larval breeding sites (natural, peri-domestic and domestic) and positive sites (with at least one *Aedes* larvae or pupae). Immature stages were collected from positive sites, recorded, transported in insectaries and reared to adult stage for identification.

Mosquitoes identified as *Ae. albopictus* were stored in individual tubes containing a desiccant at −20°C for further molecular analysis.

### Entomological indexes

The level of *Ae. aegypti* and *Ae. albopictus* infestation was assessed from standard indexes based on immature stages, including the house index (percentage of houses positive for larvae and/or pupae) and the Breteau index (number of positive containers per 100 houses). Additional indexes based on the presence or absence and the number of larvae or pupae were also used, including the larvae index (number of larvae L3-4 per 100 houses) and the pupae index (number of pupae per 100 houses) [20]. The productivity of a container type was defined as the number of L3-4 or pupae in each divided by the total number of L3-4 or pupae in all container types [38]. The larvae (L3-4) per person index and the pupae per person index were also estimated [39].

### Statistical analysis

All statistical analyses were performed in STATA version 11 (StataCorp College Station, Texas 77845). The distribution of each variable was observed. The type of container, water turbidity, the presence of vegetal debris inside the container, the presence of vegetation around the container, sun exposure and the presence of any immature stage of *Ae. albopictus* and *Ae. aegypti* were defined as categorical variables and expressed as percentages. The effect of each variable on the presence of vectors was examined in the chi-square or Fisher exact test. Numerical variables (container volume, volume of water inside the container, number of L3-4 and pupae) were described as means and standard deviations and compared in the Student t test or the Kruskal-Wallis test when the Student t test was not appropriate. Contingency tables were generated and the relation between container characteristic and presence or absence of L3–4 and pupae (immature stage) of *Ae. aegypti* or *Ae albopictus* was analysed using chi-square (or Fisher exact test if appropriate). A *p* value <0.05 was considered significant. In a second step, the presence or absence of immature stages was analysed by binary logistic regression with a conditional backwards stepwise procedure. The potential predictors tested corresponded to the main larval habitat characteristics described above. A test of correlation was also performed to determine the relations between numbers of L3–4 and pupae of *Ae. aegypti* or *Ae. albopictus* and certain breeding site characteristics, such as the container volume, volume of water inside the container, distance to the nearest building and distance of the container to plants. The GPS coordinates of houses surveyed and positive larval habitats of the two species were projected onto maps with ArcGis software (ArcGis®9.2, ESRI).

### Mitochondrial DNA analysis for *Ae. albopictus*

Sequence polymorphisms in the mitochondrial genes encoding for the nicotinamide adenine dinucleotide dehydrogenase subunit 5 (*ND5*) and for cytochrome oxidase I (*COI*) were explored in 95 individual *Ae. albopictus* mosquitoes from CAR. We included in the analysis 22 specimens of *Ae. albopictus* from Franceville (1°37′S, 13°34′E) and Dienga (1°52′S, 12°40′E) in Gabon, collected at the larval stage in June 2013. DNA extraction and PCR amplification were done as described previously [34]. Mosquito DNA extracts were used as templates to amplify a 400-bp fragment of *ND5* and a 550-bp fragment of *COI*. PCR products were purified and sent to GATC Biotech (Konstanz, Germany) for sequencing. Sequences were cleaned, when necessary, with SEQSCAPE software 2.5 (Applied Biosystems) and aligned with Clustal W [40]. *ND5* and *COI* sequences were numbered according to the reference sequences GeneBank ID JF309321 and JF309317, respectively. Basic sequence statistics, including the number of haplotypes per sample, the number of segregating sites (S), haplotype diversity, nucleotide diversity (π) and the average number of nucleotide differences, were computed with DnaSP 4.10.9 [41]. The statistical tests of Tajima [42], Fu and Li [43] and Fu [44] were used with DnaSP to test non-neutral evolution and deviation from mutation-drift equilibrium. The phylogenetic relations between *COI* and *ND5* haplotypes recorded in CAR and previously published sequences (Table S1) of *Ae. albopictus* from Asia, the Americas, the Indian Ocean, Europe and central Africa were explored by Bayesian inference analysis. MrModeltest v2.2 [45] was first used to select the model that best fit the *ND5* and *COI* nucleotide sequence data (under Akaike's information criterion). Analyses were performed with MrBayes 3.1.2 [46], and four Markov chains were run for 200 000 generations (sampling every 10 generations) to allow adequate time for convergence. The first 50 000 resulting trees were discarded as burn-in, and the remaining 150 000 sampled trees were used to estimate the 50% majority rule consensus tree and the Bayesian posterior probabilities. All Markov chain Monte Carlo runs were repeated twice to confirm consistent approximation of the posterior parameter distributions.

## Results

### Pre-imaginal infestation

We investigated 354 houses in 34 clusters or quarters in Bangui, with 3855 inhabitants. Of 176 potential larval development sites investigated early in the wet season, 52 (29.5%) contained immature stages of *Ae. aegypti* and/or *Ae. albopictus*. Late in the wet season, 97 of 209 potential larval habitats surveyed (46.4%) were positive. Several other mosquito species were found with *Ae. aegypti* and *Ae. albopictus* at both surveys: *Anopheles gambiae* s.l. Giles 1902, *Culex quinquefasciatus* Say 1823, *Culex perfuscus* Edwards 1914, *Culex tigripes* De Grandpré & De Charmoy 1900.

Early in the wet season, all the larval infestation indexes calculated for *Ae. aegypti* were significantly higher (*p*<10^−3^, chi-square test) than those for *Ae. albopictus*, except the house and Breteau indexes, for which no significant difference was found. In contrast, higher infestation with *Ae. albopictus* was observed late in the wet season for all indexes except the house and Breteau indexes (Table 1). The proportion of containers infested by *Ae. albopictus* only was significantly higher late rather than early in the wet season (*p*<0.05, Fisher exact test), whereas no significant difference was found in the proportion of containers infested by *Ae. aegypti* only early and late in the wet season (Table 2). The proportion of containers infested by *Ae. aegypti* with or without *Ae. albopictus* and by *Ae. albopictus* with or without *Ae. aegypti* were 98.5% and 86.5% in the early wet season and 87.6% and 88.6% in the late wet season, respectively (data not shown). No statistically significant difference in the proportions of containers infested by *Ae. aegypti* with or without *Ae. albopictus* and *Ae. albopictus* with or without *Ae. aegypti* was found in any collection period, suggesting that infestation of containers by these species is comparable, irrespective of the season.

**Table 1:** Infestation indexes of immature stages of *Aedes aegypti* and *Ae. albopictus* in Bangui.

		Early wet season						Late wet season				
Species	House index	Breteau index	Larvae index	Pupae index	Larvae per person index	Pupae per person index	House index	Breteau index	Larvae index	Pupae index	Larvae per person index	Pupae per person index
*Ae. aegypti*	9.03	14.4	768.6	99.1	0.7	0.1	21.8	16.5	913.4	84.4	0.8	0.08
*Ae. albopictus*	7.3	12.7	437.8	52.2	0.4	0.05	21.8	16.2	1381.8	120.7	1.3	0.1
*p*	0.8	0.7	<10^−3^	<10^−3^	<10^−3^	<10^−3^	1	0.9	<10^−3^	<10^−3^	<10^−3^	<10^−3^

**Table 2:** Container type of *Ae. aegypti* and *Ae. albopictus* in Bangui during early and late rainy season.

	Early wet season					Late wet season				
Type of container	n inspected	% positive	% *Ae. aegypti* only	% *Ae. albopictus* only	% mixed	n inspected	% positive	% *Ae. aegypti* only	% *Ae. albopictus* only	% mixed
	n = 176	n = 52	n = 7	n = 1	n = 44	n = 209	n = 97	n = 11	n = 12	n = 74
**Domestic**	**31**	**17.3**	**14.3**	**0.0**	**18.2**	**18**	**8.2**	**0.0**	**8.3**	**9.4**
Watering place*	6	0.0	0.0	0.0	0.0	5	1.0	0.0	0.0	1.4
Water storage	11	0.0	0.0	0.0	0.0	5	1.0	0.0	0.0	1.4
Flower pots	14	17.3	14.3	0.0	18.2	8	6.2	0.0	8.3	6.7
**Peri-domestic**	**143**	**80.8**	**71.4**	**100.0**	**81.8**	**189**	**90.8**	**100.0**	**91.7**	**89.2**
Used tyres	49	51.9	42.9	100.0	50.0	79	46.4	45.5	25.0	50.0
Discarded tanks	63	28.8	28.6	0.0	31.8	82	30.0	45.5	50.0	24.3
Miscellaneous	31	0.0	0.0	0.0	0.0	28	14.4	9.0	16.6	14.9
**Natural**	**2**	**1.9**	**14.3**	**0.0**	**0.0**	**2**	**1.0**	**0.0**	**0.0**	**1.4**

n inspected, number of potential containers inspected; % positive, percentage of containers infested with at least one larva or pupa of one species; *Ae. aegypti* only, containers containing only *Ae. aegypti*; *Ae. albopictus* only, containers containing only *Ae. albopictus*; mixed, containers infested with at least one larva or pupa of each species;

* Container used to give drinking-water to pets.

### Container occupancy by *Ae. aegypti* and *Ae. albopictus*

**Figure 2:**
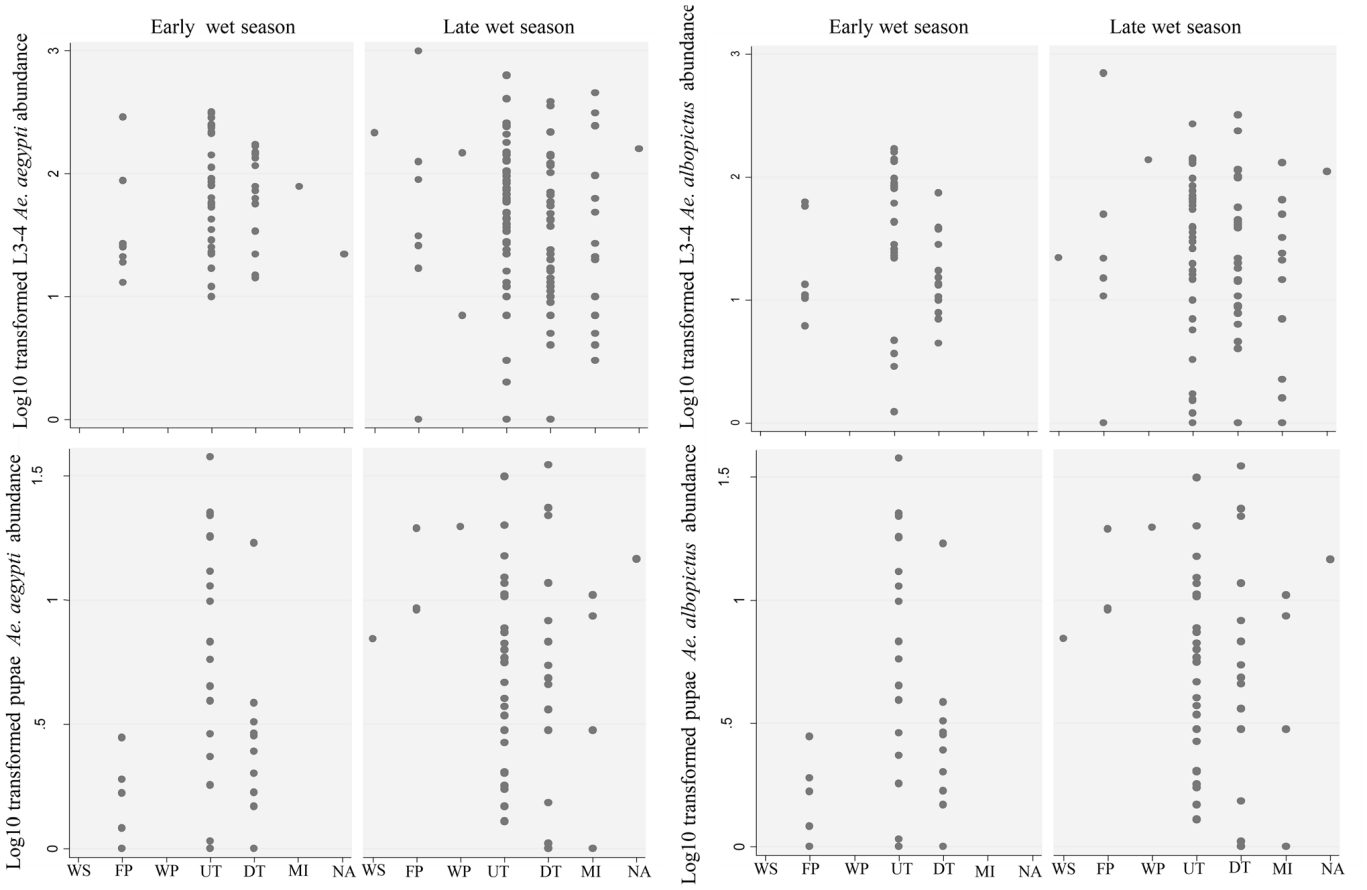
Total abundance of immature stages of *Aedes aegypti* and *Ae. albopictus* per container. Each two-letter abbreviation on the x-axis corresponds to a type of container as follows: WS, water storage; FP, flower pot; WP, watering place; UT, used tyres; DT, discarded tanks; MI, miscellaneous; NA, natural.

During entomological surveys in both periods, all three defined categories of container were found: domestic (watering place, water storage and flower pots), peri-domestic (used tyres, discarded tanks, miscellaneous) and natural containers (leaf axils of *Colocasia* spp. taro plants). Peri-domestic containers represented the main infested container type in both periods, with a prevalence of infestation of 80.8% and 90.8%, respectively (Table 2). The most productive containers for both species during the two periods of investigation were used tyres, although the distribution of larvae (L3–4) was over-dispersed early in the wet season (Figure 2). The domestic containers were more likely to contain larvae early (17.3%) than late in the wet season (8.2%) (*p*<0.05, chi-square test), flower pots being the most productive domestic containers.

We used a binary logistic regression model to test the association between container characteristics and the presence of immature stages of *Ae. aegypti* and *Ae. albopictus*. Multivariate analyses showed that early in the wet season the presence of the two species was significantly associated with the type of container (used tyres or flower pots), the presence of plant debris inside the container and the presence of vegetation in the vicinity of containers (for *Ae. aegypti* only), whereas late in the wet season, only the presence of vegetation around the potential containers was significantly associated with the presence of the two species (Table 3).

**Table 3:** Container characteristics associated with the presence of immature stages of *Ae. albopictus* and *Ae. aegypti* in Bangui.

	Early wet season							Late wet season						
		*Aedes albopictus*			*Aedes aegypti*				*Aedes albopictus*			*Aedes aegypti*		
Category	Number	%	Univariate, OR (CI 95%)	Multivariate, OR (CI 95%)	%	Univariate, OR (CI 95%)	Multivariate, OR (CI 95%)	Number	%	Univariate, OR (CI 95%)	Multivariate, OR (CI 95%)	%	Univariate, OR (CI 95%)	Multivariate, OR (CI 95%)
**Type of container**														
Water storage	11	0	Reference	Reference	0	Reference	Reference	5	20	Reference	Reference	20	Reference	NA
Flower pot	14	57.1	5.8 (2.6–12.7)*	6.1 (1.6–23.6)*	64.3	6.3 (2.9–13.6)*	9.1 (2.4–34.9)*	8	75	2.1 (1.1–3.7)*	0.5 (0.07–3.8)	62.5	3.8 (0.8–16.8)	NA
Watering place	6	0	Reference	Reference	0	Reference	Reference	5	20	Reference	Reference	20	Reference	Reference
Used tires	49	44.9	9.4 (2.8–31.2)*	3.9 (1.6–9.8)*	51	10.9 (3.2–36.7)*	4.7 (1.9–11.6)*	79	49.4	6.4(1.2–33.1)*	0.3 (0.09–0.8)	48.1	2.1 (1.2–3.8)*	0.3 (0.1–1.0)
Discarded tanks	63	22.2	Reference	Reference	23.8	Reference	Reference	85	29.4	Reference	Reference	28.2	Reference	Reference
Miscellaneous	31	0	Reference	Reference	0	Reference	Reference	25	44	Reference	Reference	40	Reference	Reference
Natural	2	0	Reference	Reference	50	Reference	Reference	2	50	Reference	Reference	50	Reference	Reference
**Sun exposure**														
Yes	82	13.4	Reference	Reference	14.6	Reference	Reference	44	45.5	1.3 (0.9–2.8)	NA	45.5	1.4 (0.7–2.8)	NA
No	94	35.1	3.5 (1.6–7.5)*	0.5 (0.2–1.3)	40.4	3.9 (1.9–8.3)*	0.3 (0.1–0.9)	165	38.8	Reference	NA	36.4	Reference	NA
**Nature of water**														
Clear	133	22.6	Reference	NA	24.1	Reference	Reference	170	40.6	Reference	NA	36.5	Reference	NA
Turbid	44	31.8	0.6 (0.3–1.3)	NA	40.9	2.2 (1.1–4.5)*	1.4 (0.5–3.4)	37	37.8	0.9 (0.4–1.8)	NA	45.9	1.5 (0.7–3.0)	NA
Polluted	9	0	NA	NA	0	NA	NA	11	45.5	1.2 (0.3–4.1)	NA	45.5	NA	NA
**Plant debris inside the container**														
Yes	87	46	18.1 (6.1–53.7)*	8.9 (2.7–29.5)*	49.4	11.4 (4.7–27.6)*	4.1 (1.5–11.3)*	117	45.3	1.6 (0.9–2.8)	NA	45.3	2.0 (1.1–3.5)	1.5 (0.8–3.0)
No	89	4.5	Reference	Reference	7.9	Reference	Reference	92	33.7	Reference	NA	29.3	Reference	Reference
**Vegetation around the container**														
Yes	64	40.6	3.6 (1.7–7.3)*	2.0 (0.8–4.8)	48.4	4.6 (2.3–9.2)*	3.3 (1.4–8.0)*	80	67.5	6.8 (3.7–12.7)*	16.7 (5.5–51.0)*	63.8	6.0 (3.3–11.2)*	12.7 (4.5–36.0)*
No	112	16.1	Reference	Reference	17	Reference	Reference	129	23.3	Reference	Reference	22.5	Reference	Reference

OR, odds ratio; CI, confidence interval;

* significant association;

NA, not applicable; Reference, ‘comparator group’ for estimating the OR.

We also explored the correlation between numbers of larvae and pupae of *Ae. aegypti* and *Ae. albopictus* and breeding site characteristics, such as distance of a container from a building and from plants, container volume and water volume. Early in the wet season, the distance of a container from plants was significantly inversely correlated with the number of larvae of both species (correlation coefficient (*r*) = −0.15, *p*<0.05 for *Ae. aegypti*; *r* = −0.18, *p*<0.02 for *Ae. albopictus*) and the number of pupae (*r* = −0.20, *p*<0.01 for *Ae. aegypti*; *r* = −0.20, *p*<0.01 for *Ae. albopictus*), whereas late in the wet season no significant correlation was found between container characteristics and productivity.

### Spatial distribution of immature stage of *Aedes* spp

**Figure 3:**
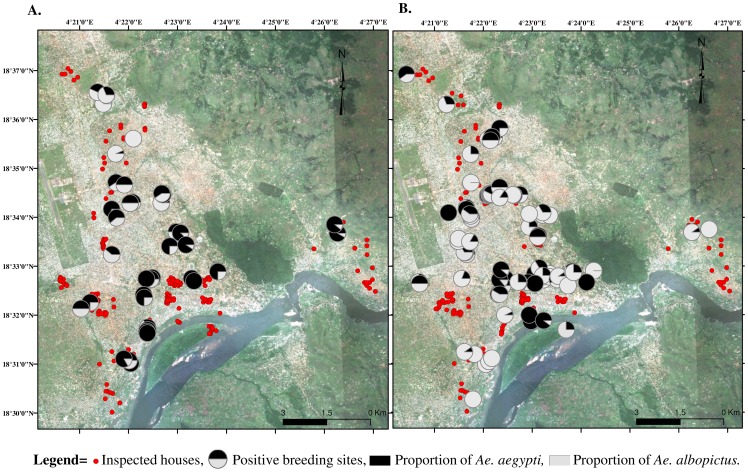
Spatial distribution of surveyed houses and positive larval habitats of *Aedes* spp. in Bangui. The surveys were conducted during the early wet season (A) and the late wet season (B).

In the 52 positive larval habitats identified early in the wet season, 3556 specimens of immature stages of *Aedes* spp. were identified, 60% of which were *Ae. aegypti* and 40% *Ae. albopictus*. In contrast, late in the wet season, 4250 specimens of *Aedes* spp. were recorded in 97 positive containers, of which 36% corresponded to *Ae. aegypti* and 64% to *Ae. albopictus*. These data suggest that *Ae. aegypti* is more prevalent early in the wet season and *Ae. albopictus* late in the wet season. The spatial distribution (Figure 3) of the two species showed that *Ae. aegypti* mosquitoes occur throughout Bangui early in the wet season, whereas *Ae. albopictus* is present in almost all environments late in the wet season, suggesting efficient expansion of this species, which appeared to be most prevalent in suburban areas. No trend in segregation of the species according to unplanned or planned environment was found (*p*>0.05, data not shown).

### Distribution of *Ae. aegypti* and *Ae. albopictus* in southern CAR

Surveys in six additional locations in southern CAR during the late wet season showed that the two species coexisted and often shared the same larval habitats, except at Bouar, where *Ae. aegypti* was found alone. *Ae. albopictus* was the more prevalent in all localities in which both species were found (Table 4).

**Table 4:** Immature mosquito sampling in southern Central African Republic.

Locality	Period of sampling	Type of container inspected	Containers inspected	Positive containers	Container with *Ae. aegypti* only	Container with *Ae. albopictus* only	Mixed	Number of *Ae. albopictus* identified (%)	Number of *Ae. aegypti* identified (%)
Mbaïki	October 2012	Used tyres, discarded tanks	18	7	0	6	1	256 (96.6)	9 (3.4)
Batalimo	October 2012	Used tyres, discarded tanks, tree holes	8	6	1	3	2	156 (98.1)	3 (1.9)
Mongoumba	October 2012	Watering place, discarded tanks	13	4	0	0	2	25 (89.3)	3 (10.7)
Boda	November 2012	Used tyres, earthen jar	11	5	0	4	1	86 (98.9)	1 (1.1)
Berberati	November 2012	Car wrecks, discarded tanks, tin cans	23	6	0	0	6	266 (66.8)	132 (33.2)
Bouar	November 2012	Used tyres	11	6	6	0	0	0	511 (100)

### Mitochondrial DNA analysis of *Ae. albopictus*

Nucleotide sequences of the mtDNA *ND5* gene were retrieved from 91 specimens originating from six localities in CAR. Complete overlap of all the fragments spanned 399 nucleotides, of which two were polymorphic, defining three haplotypes, resulting in low haplotype and nucleotide indexes. The most frequent haplotype, H1 (89%), was detected in all geographical samples (Tables 5 and 6).

**Table 5:** MtDNA *COI* and *ND5* haplotypes recorded in *Ae. albopictus* in the Central African Republic.

	*ND5*			*COI*	
		2 2 3			3 3 3
		0 6 8			2 0 4 6
Haplotype*	Frequency	8 8 5	Haplotype	Frequency	4 6 5 6
Ref. [JF309321]		A T T	Ref. [JF309317]		T G T C
H1 [KC979137]	103	. . .	H1 [KC979140]	86	. . . .
H2 [KC979138]	5	G . A	H2 [KC979141]	4	C . . .
H3 [KC979139]	5	. C A	H3 [KC979142]	1	. A . .
			H4 [KC979143]	1	. A A T

Only polymorphic positions are shown and are numbered with reference (Ref) to the published *Ae. albopictus* sequences for *ND5* (JF309321; Cameroon) and *COI* (JF309317; Cameroon). Dots represent identity with respect to the reference.

* GenBank accession number in brackets.

Frequency, number of times the haplotype was found in the total sample.

**Table 6:** Summary statistics for mtDNA gene polymorphism in *Ae. albopictus* in the Central African Republic.

Locality	N	Mt gene	Hp	S	HpD	π	K	D	D*	F*	Fs
Berberati	12	*ND5*	H1, H2, H3	2	0.53	0.0014	0.57	−0.38	−0.37	−4.42	−0.36
	10	*COI*	H1, H2	2	0.35	0.0008	0.35	0.01	0.80	0.68	0.41
Boda	6	*ND5*	H1	0	0.00	0.0000	NC	NC	NC	NC	NC
	6	*COI*	H1	0	0.00	0.0000	NC	NC	NC	NC	NC
Mongoumba	8	*ND5*	H1	0	0.00	0.0000	NC	NC	NC	NC	NC
	6	*COI*	H1	0	0.00	0.0000	NC	NC	NC	NC	NC
Batalimo	22	*ND5*	H1, H3	1	0.24	0.0008	0.24	−0.17	0.63	0.47	0.30
	10	*COI*	H1, H2, H3	2	0.37	0.0009	0.40	−1.40	−1.58	−1.71	−1.16
Mbaïki	10	*ND5*	H1, H2	1	0.35	0.0008	0.35	0.01	0.80	0.68	0.417
	9	*COI*	H1, H2	1	0.22	0.0005	0.22	−1.08	−1.18	−1.28	−0.26
Bangui	33	*ND5*	H1, H3	1	0.06	0.0000	0.06	−1.14	−1.71	−1.78	−1.29
	29	*COI*	H1, H4	3	0.07	0.0005	0.26	−1.73	−2.66*	−2.77*	0.16
Overall	91	*ND5*	H1, H2, H3	2	0.20	0.0005	0.21	−0.73	0.69	0.29	−1.01
	70	*COI*	H1, H2, H3, H4	4	0.16	0.0005	0.22	−1.54	−1.33	−1.64	−2.51

N, number of sequences analysed; Hp, number of haplotypes; S, number of segregating sites; HpD, haplotype diversity; π, nucleotide diversity; K, average number of nucleotide differences; D, Tajima statistic; D* and F*, Fu and Li statistics; Fs, Fu statistic; NC, not computed;

* *p*<0.05.

Sequences of the mtDNA *COI* gene were obtained from 70 specimens. Complete overlap of all fragments spanned 426 nucleotides, of which four were polymorphic (overall nucleotide diversity, π = 0.0005), defining four distinct haplotypes. Haplotype I dominated (91%) and was encountered in all localities (Tables 5 and 6). Sequences of mtDNA from the *COI* and *ND5* genes were also retrieved from 22 *Ae. albopictus* specimens from two localities in Gabon. The analysis revealed the existence of only one haplotype (H1) for each gene (Table 5). The sequence of the main CAR and Gabon *ND5* (H1) and *COI* (H1) haplotypes perfectly matched the dominant haplotypes found in Cameroonian *Ae. albopictus* samples from a database (Table 5). When all the sequences were analysed as a unique sample, the Tajima D, Fu and Li F* and D*, and Fu Fs statistics for the *COI* gene were negative but not statistically significant so (Table 6). Negative values for these indexes indicate an excess of rare polymorphisms in a population and suggest either population expansion or background selection [43].

**Figure 4:**
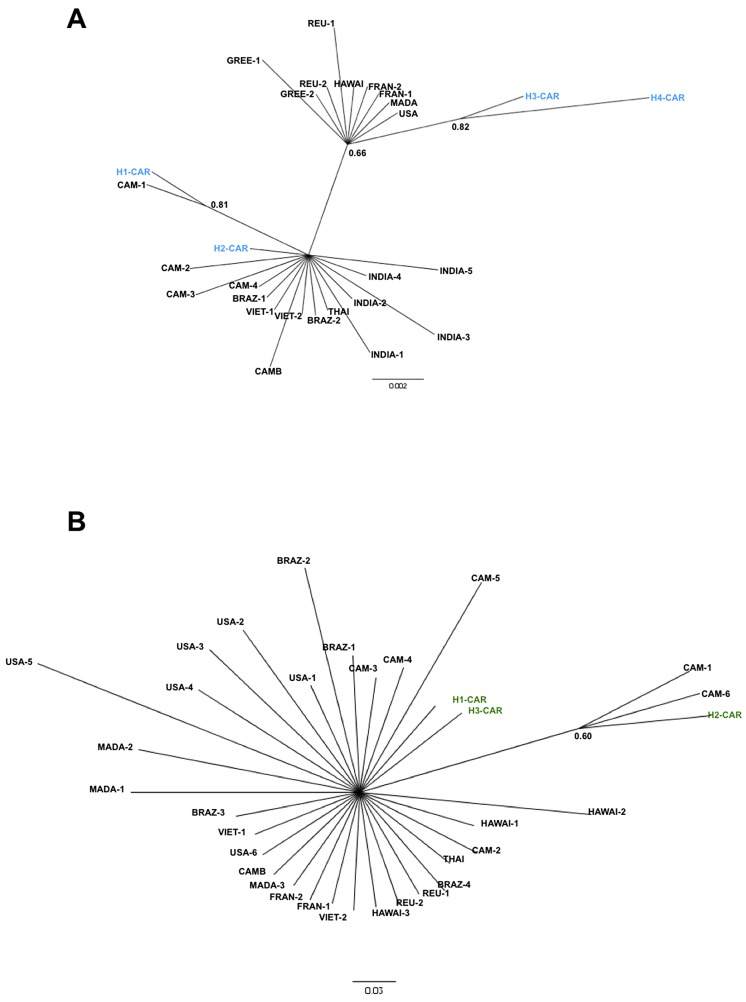
Bayesian inference hypothesis of *Ae. albopictus* phylogeny based on *COI* (A) and *ND5* (B) sequence data. The phylogeny was constructed with MrBayes 3.1.2, ngen = 2 000 000. Best-fitting models selected with the MR model test (under AIC) were HKY for *COI* and HKY+I+G for the *ND5* nucleotide datasets. Branch support is indicated by the posterior probability values. Accession numbers of *COI* and *ND5* out-group sequences are given in supporting information file Table S1.

In order to determine the geographical origin of the *Ae. albopictus* populations that are invading CAR, the phylogenetic relations between *COI* and *ND5* sequences recorded in CAR and previously published sequences were assessed by Bayesian inference. The *COI* sequences segregated into two lineages (Figure 4). The first encompassed specimens from tropical areas (Brazil, Cambodia, India, Thailand and Viet Nam), including all the Cameroonian haplotypes and two haplotypes from CAR (H1-CAR and H2-CAR). The second lineage encompassed temperate and subtropical areas (France, Greece, Madagascar, Reunion and the USA) and, surprisingly, two haplotypes from CAR (H3-CAR and H4-CAR). All the sequences were monophyletic at *ND5*.

## Discussion

This detailed study suggests that *Ae. aegypti* is most prevalent in the early wet season and *Ae. albopictus* in the late wet season. Used tyres were the most productive container for both species, independently of season. In the survey across southern CAR in the late wet season, *Ae. albopictus* was the dominant species at all sites except Bouar.

In Bangui, we found significant differences in infestation rates by larval *Ae. aegypti* and *Ae. albopictus* according to season. The *Ae. aegypti* indexes were significantly higher than those for *Ae. albopictus* in the early wet season, with the opposite situation in the late wet season, suggesting lower abundance of *Ae. albopictus* in the early and higher abundance in the late wet season. These findings are consistent with those of studies in southern Florida, USA [47]. Although both species have desiccant-resistant eggs, Juliano et al. [48] showed that *Ae. aegypti* eggs are more tolerant to high temperatures than those of *Ae. albopictus*. This would explain why resident *Ae. aegypti* is more prevalent than invasive *Ae. albopictus* in the early wet season (i.e. the warmer season) in locations where the two species are sympatric.

The larvae of both species preferentially colonized peri-domestic containers, especially used tyres and discarded tanks, irrespective of the collection period. In agreement with observations made in Cameroon [9], [18], [20], peri-domestic containers represented the bulk of the containers infested by *Ae. aegypti* or *Ae. albopictus*, thus differing from the situation in other parts of the world, particularly in Asia, where domestic containers such as water storage tanks were most commonly infested with *Ae. aegypti* [38], [49]. In many sub-Saharan towns, unplanned urbanization and lack of waste management lead to widespread water collection, thus favouring the proliferation of *Aedes* spp. The two species studied here breed in the same type of container, with a preference for used tyres, flower pots, containers with plant debris and vegetation surrounding the container early in the wet season; late in the wet season, only larval habitats surrounded by vegetation were significantly associated with the presence of immature stages of *Aedes* spp. Micro-environmental factors therefore affect the presence of larval stages in breeding sites. In addition, used tyres were found to be the most productive containers for larvae and pupae in both sampling periods. Both species are native to the forest and breed mainly in natural tree holes, which share the characteristics of tyres, as the dark colour and the dark interior provides an attractive resting or oviposition site for *Aedes* spp. The presence of plant debris inside a larval habitat can serve as a food source or a micro-habitat to hide and avoid predators [50]. Surrounding vegetation can provide shade to reduce the water temperature of the larval habitat [51]. The association of the two species with the same micro-environmental conditions suggests that the invasive species, *Ae. albopictus*, shares the ecological niche of the resident species, *Ae. aegypti*. Competition for resources will, however, lead to segregation of habitats according to macro-environmental variations such as urban environmental gradients, as shown by other authors [7], [21], [51], or reduction of abundance of the indigenous species [25], [26]. In addition, recent work shows that the two species are able to mate in nature and that *Ae. albopictus* males effectively sterilize *Ae. aegypti* females [52], [53]. The authors suggest that this form of mating interference, called satyrization, could explain the competitive displacement of resident *Ae. aegypti* by the invasive *Ae. albopictus* where they co-occur.

The invasive species *Ae. albopitus* was more prevalent in all the sites investigated, except in Bouar, where only *Ae. aegypti* was found. This suggests rapid spread and good adaptation of *Ae. albopictus* in CAR. Previous surveys reported the presence of this species only in Bangui and Bayanga [12], [54], at lower proportions (container index below 5) than observed in this study. The low density of *Ae. albopictus* reported in 2010 prompted Diallo et al. [12] to propose that its introduction is recent, probably through migratory flow and trade between the CAR and neighbouring countries, especially Cameroon, where the species was recorded for the first time in central Africa in 2000 [9]. Bouar is located near Cameroon at 6°N latitude, beyond which *Ae. albopictus* has not been found. This observation is consistent with studies in Cameroon, which suggest that the northern limit of *Ae. albopictus* invasion in Africa is around 6°N [18], [20].

The higher prevalence of *Ae. albopictus* at all the sites investigated is in agreement with the findings of studies in other central African countries (Cameroon and Gabon), which suggest a dominance of the invasive species over the indigenous species in sites where the two species co-exist [1], [14], [18], [20]. A decrease in indigenous *Ae. aegypti* after invasion by *Ae. albopictus* was also suspected in several localities in the Indian Ocean, such as Mayotte and Reunion [27], [28], [29]. In addition, invasive species have a competitive advantage over native species or first established invasive species, as observed in Brazil and south-eastern USA, where established invasive *Ae. aegypti* were displaced by recently invading *Ae. albopictus* [25], [26], and in Asia, where *Ae. aegypti* has an overall competitive advantage over *Ae. albopictus*, especially in urban areas [31], [55].

MtDNA markers have been used extensively to assess the genetic diversity of *Ae. albopictus* populations across most of its geographical range. The degree of polymorphism found in *ND5* and *COI* sequences in this study was low (three haplotypes for *ND5* and four for *COI*), consistent with previous studies of populations sampled in newly invaded areas [32], [33], [34], [56], [57], in which the number of haplotypes per country never exceeded five, regardless of the mtDNA marker used (*ND5*, *COI* or *Cytb*). In CAR, the low overall mtDNA diversity is consistent with recent introduction of a few founder females, as suggested by Diallo et al. [12], or may be related to ubiquitous *Wolbachia* infection in populations of this species, as suggested by Armbruster et al. [58]. Analyses of *COI* sequences revealed that central African *Ae. albopictus* are partly related to a tropical lineage (H1-CAR and H2-CAR) and partly to a temperate or subtropical lineage (H3-CAR and H4-CAR), although H3 and H4 for COI are represented by only one specimen each. These results suggest that the populations present in CAR are derived from multiple invasions and multiple population sources. It is likely that Cameroon, which shares a border with CAR, was the main source of the invasion. Nevertheless, our previous study in Cameroon indicated that *Ae. albopictus* is related only to a tropical lineage, such as H1-CAR and H2-CAR haplotypes, suggesting that haplotypes H3-CAR and H4-CAR were introduced independently, from a temperate or a subtropical source, and make a minor contribution to the invasion in CAR. As CAR is landlocked, with no direct access to the sea, introduction of this species could have been by air with the transport of logistical equipment by foreign armed forces or the ubiquitous nongovernmental organizations.

The high infestation indexes of both species (particularly of *Ae. albopictus*) suggest an imminent risk for large outbreaks of arbovirus infections such as dengue and chikungunya in CAR, as in Cameroon in 2006 [13] and Gabon in 2007 [59] and 2010 [14], where the species was identified as or suspected to be the main vector. *Ae. albopictus* was also suspected of being responsible for transmission of chikungunya virus during the large outbreak in the Republic of Congo in 2011 [16].

As the dynamics of epidemics are correlated with the seasonal dynamics of vector populations [60], additional sampling, covering additional locations and spanning several seasons, would be beneficial. Nevertheless, our data on the spatial distribution, container type and productivity of larval development sites provide a useful basis for planning vector control programmes.

## Supporting Information

Table S1 Phylogenetic relations between *COI* and *ND5* haplotypes recorded in the Central African Republic and previously published sequences of *Ae. albopictus* from Asia, the Americas, the Indian Ocean, Europe and central Africa.
(DOC)
