# Supplementary material for: Temporal Patterns of Abundance of Aedes aegypti and Aedes albopictus (Diptera: Culicidae) and Mitochondrial DNA Analysis of Ae. albopictus in the Central African Republic
Source: PLoS Negl Trop Dis. 2013 Dec 12;7(12):e2590. doi: 10.1371/journal.pntd.0002590 (PMC3861192; doi:10.1371/journal.pntd.0002590)
Supplement: Table S1 — Phylogenetic relations between COI and ND5 haplotypes recorded in the Central African Republic and previously published sequences of Ae. albopictus from Asia, the Americas, the Indian Ocean, Europe and central Africa. (DOC) [file pntd.0002590.s001.doc]

| **mtDNA Marker** | **Geographical Area** | **Outgroup Name** | **Country** | **Climate** | **Accession Number** | **Authors** |
| --- | --- | --- | --- | --- | --- | --- |
| **COI** | **Europe, West** | FRAN-1 | France | Temperate | AJ971008 | Mousson et al., 2005 |
|  |  | FRAN-2 | France | Temperate | AJ971009 | Mousson et al., 2005 |
|  |  | GREE-1 | Greece | Temperate | AY748238 | Patsoula et al., 2006 |
|  |  | GREE-2 | Greece | Temperate | AY748239 | Patsoula et al., 2006 |
|  | **America, North** | USA | United States of America | Temperate | AJ971005 | Mousson et al., 2005 |
|  | **Pacific Ocean, North** | HAWAI | Hawaï (USA) | Sub-Tropical | AJ971011 | Mousson et al., 2005 |
|  | **Indian Ocean, South West** | MADA | Madagascar | Sub-Tropical | AJ971007 | Mousson et al., 2005 |
|  |  | REU-1 | La Réunion Island | Sub-Tropical | AJ971012 | Mousson et al., 2005 |
|  |  | REU-2 | La Réunion Island | Sub-Tropical | AJ971013 | Mousson et al., 2005 |
|  | **Africa, Central** | CAM-1 | Cameroon | Tropical | JF309317 | Kamgang et al., 2011 |
|  |  | CAM-2 | Cameroon | Tropical | JF309318 | Kamgang et al 2011 |
|  |  | CAM-3 | Cameroon | Tropical | JF309319 | Kamgang et al., 2011 |
|  |  | CAM-4 | Cameroon | Tropical | JF309320 | Kamgang et al., 2011 |
|  | **Asia, South** | INDIA-1 | India | Tropical | AY729984 | Kumar et al., 2007 |
|  |  | INDIA-2 | India | Tropical | AY834241 | Mousson et al., 2005 |
|  |  | INDIA-3 | India | Tropical | DQ310142 | Kumar et al., 2007 |
|  |  | INDIA-4 | India | Tropical | DQ424959 | Kumar et al., 2007 |
|  |  | INDIA-5 | India | Tropical | EU250306 | Dai, unpublished |
|  | **America, South** | BRAZ-1 | Brazil | Tropical | AJ971003 | Mousson et al., 2005 |
|  |  | BRAZ-2 | Brazil | Tropical | AJ971014 | Mousson et al., 2005 |
|  | **Asia, South-East** | CAMB | Cambodia | Tropical | AJ971006 | Mousson et al., 2005 |
|  |  | THAI | Thaïland | Tropical | AJ971015 | Mousson et al., 2005 |
|  |  | VIET-1 | Vietnam | Tropical | AJ971004 | Mousson et al., 2005 |
|  |  | VIET-2 | Vietnam | Tropical | AJ971010 | Mousson et al., 2005 |
| **ND5** | **Europe, West** | FRAN-1 | France | Temperate | AJ971021 | Mousson et al., 2005 |
|  |  | FRAN-2 | France | Temperate | AJ971022 | Mousson et al., 2005 |
|  | **America, North** | USA-1 | United States of America | Temperate | AY049970 | Birungi and Mustermann, 2002 |
|  |  | USA-2 | United States of America | Temperate | AY049972 | Birungi and Mustermann, 2002 |
|  |  | USA-3 | United States of America | Temperate | AY049971 | Birungi and Mustermann, 2002 |
|  |  | USA-4 | United States of America | Temperate | AY049973 | Birungi and Mustermann, 2002 |
|  |  | USA-5 | United States of America | Temperate | AY049974 | Birungi and Mustermann, 2002 |
|  |  | USA-6 | United States of America | Temperate | AJ971018 | Mousson et al., 2005 |
|  | **Pacific Ocean, North** | HAWAI-1 | Hawaï (USA) | Sub-Tropical | EU118296 | Usmani-Brown et al., 2009 |
|  |  | HAWAI-2 | Hawaï (USA) | Sub-Tropical | EU118297 | Usmani-Brown et al., 2009 |
|  |  | HAWAI-3 | Hawaï (USA) | Sub-Tropical | AJ971024 | Mousson et al., 2005 |
|  | **Indian Ocean, South West** | MADA-1 | Madagascar | Sub-Tropical | AY049976 | Birungi and Mustermann, 2002 |
|  |  | MADA-2 | Madagascar | Sub-Tropical | AY049975 | Birungi and Mustermann, 2002 |
|  |  | MADA-3 | Madagascar | Sub-Tropical | AJ971020 | Mousson et al., 2005 |
|  |  | REU-1 | La Réunion Island | Sub-Tropical | AY785426 | Fernandez et al. unpublihed |
|  |  | REU-2 | La Réunion Island | Sub-Tropical | AY785425 | Fernandez et al. unpublihed |
|  | **Africa, Central** | CAM-1 | Cameroon | Tropical | EU118294 | Usmani-Brown et al., 2009 |
|  |  | CAM-2 | Cameroon | Tropical | EU118295 | Usmani-Brown et al., 2009 |
|  |  | CAM-3 | Cameroon | Tropical | JF309321 | Kamgang et al. 2011 |
|  |  | CAM-4 | Cameroon | Tropical | JF309322 | Kamgang et al.,2011 |
|  |  | CAM-5 | Cameroon | Tropical | JF309323 | Kamgang et al., 2011 |
|  |  | CAM-6 | Cameroon | Tropical | JF309324 | Kamgang et al., 2011 |
|  | **America, South** | BRAZ-1 | Brazil | Tropical | AY049968 | Birungi and Mustermann, 2002 |
|  |  | BRAZ-2 | Brazil | Tropical | AY049969 | Birungi and Mustermann, 2002 |
|  |  | BRAZ-3 | Brazil | Tropical | AJ971016 | Mousson et al., 2005 |
|  |  | BRAZ-4 | Brazil | Tropical | AJ971027 | Mousson et al., 2005 |
|  | **Asia, South-East** | CAMB | Cambodia | Tropical | AJ971019 | Mousson et al., 2005 |
|  |  | THAI | Thaïland | Tropical | AJ971028 | Mousson et al., 2005 |
|  |  | VIET-1 | Vietnam | Tropical | AJ971017 | Mousson et al., 2005 |
|  |  | VIET-2 | Vietnam | Tropical | AJ971023 | Mousson et al., 2005 |
